# Inherent low Erk and p38 activity reduce Fas Ligand expression and degranulation in T helper 17 cells leading to activation induced cell death resistance

**DOI:** 10.18632/oncotarget.10913

**Published:** 2016-07-28

**Authors:** Doureradjou Peroumal, Thiruvaimozhi Abimannan, Ravichandra Tagirasa, Jyothi Ranjan Parida, Santosh Kumar Singh, Prasantha Padhan, Satish Devadas

**Affiliations:** ^1^ Infectious Disease Biology, Institute of Life Sciences, Chandrashekarpur, Bhubaneswar, Odisha, India; ^2^ Kalinga Institute of Medical Sciences, Patia, Bhubaneswar, Odisha, India; ^3^ Institute of Medical Sciences & SUM Hospital, Kalinga Nagar, Bhubaneswar, Odisha, India

**Keywords:** T helper cells, MAPK, AICD, FasL, rheumatoid arthritis, Immunology and Microbiology Section, Immune response, Immunity

## Abstract

Activation Induced Cell Death of T helper cells is central to maintaining immune homeostasis and a perturbation often manifests in aberrant T helper cells that is associated with immunopathologies. Significant presence of T cells positive for IL-17A (Th17) and dual positive for IFN-γ/IL-17A (Th1/Th17) in both effector (CD45RA^+^RO^+^) and memory (CD45RA^−^RO^+^) compartments with differential FasL protein in RA peripheral blood suggested their differential TCR AICD sensitivity. Lowered active caspase-3 in Th17 and Th1/Th17 over Th1 cells confirmed their capability to resist AICD and pointed to early upstream events. Differential MAPK activities, FasL protein and downstream caspase-3 activities in murine Th1 and Th17 cells established distinct TCR mediated signaling pathways and suggested low Erk and p38 activity as pivotal for AICD sensitivity. We extrapolated our mouse and human data and report that Fas-FasL is the preferred death pathway for both Th1 and Th17 and that inherently low Erk2 activity protected Th17 cells from TCR AICD. The presence of significantly higher numbers of aberrant T helper cells in RA also suggest an inflammatory cytokine milieu and AICD insensitive T cell link to sustained inflammation. Re sensitization to apoptosis by targeting MAPK activity especially Erk2 in RA might be of therapeutic value.

## INTRODUCTION

Rheumatoid arthritis (RA) is a severe debilitating inflammatory joint disease with unknown etiology. Epidemiologically, the prevalence is approximately 1% of the world's population and affects women three times more than men [1]. The hallmark of this disease are episodic chronic inflammation, joint swelling and progressive joint destruction mediated largely in part by inflammatory T helper cells [2]. Th1 cells amongst the then known T helper subsets and its signature cytokine IFN-γ were considered to primarily cause and sustain pathology in RA [3]. Subsequently the use of IL-12p40 and IL-12p35 KO mice to study autoimmune arthritis clearly distinguished the roles of IL-12 driven Th1 and IL-23 driven new T helper subset Th17 in initiating and exacerbating joint destruction in RA [4–6]. Th17 cells that make IL-17A/ IL-17F, IL-22, IL-21 etc. are highly inflammatory and have been shown to activate osteoclasts that lead to bone destruction in RA [7, 8] and are also involved in other autoimmune disorders [8]. Coincidentally the inability of IL-12p35 antibody to block the development of autoimmune arthritis and the exaggerated Immune pathology in IFN-γ KO suggested a dampening role for IFN-γ [9–11]. In fact these and other studies show that the absence of IFN-γ exacerbated the disease severity in many IL-17 mediated autoimmune diseases including RA [3, 8, 9] while other recent reports still suggest a role for IFN-γ due to the presence of auto antigen specific Th1 cells in RA [12]. Despite Th1's controversial role, Th17's involvement in RA is unequivocal and Th17's aberrant existence might decisively influence immune pathology.

Under physiological conditions T helper cells are important for orchestrating pathogen specific immune responses apart from maintaining immune tolerance. Antigen presentation to naïve CD4 T cells by dendritic cells along with appropriate co-stimulus and cytokines, initiates the activation of antigen specific T helper cells. Several elegant studies and models have shown that antigens drive T helper subsets' differentiation and consequent to this activation; T helper cells differentiate, proliferate (clonal expansion) and mount antigen specific immunity [13]. Th1 immunity is directed against intra cellular infections while Th17 [14, 15] are primarily directed against fungal and extra cellular bacterial infection [15–18]. It is to be noted that entirely different set of cytokines namely: IL-12 and IL-2; TGF-β, IL-1 and IL-6 drive Th1 or Th17 differentiation but remarkably both subsets die through Fas-FasL inter action [19]. This in built homeostatic mechanism ensures controlled inflammation and self-tolerance to a very large extent. However any escape of inflammatory T helper cells such as Th1, Th17 and or other T helper cells such as Th2 from T Cell Receptor Activation Induced Cell Death (TCR AICD) has pathology associated that not only challenges self-tolerance but has also been shown to cause and exacerbate autoimmune and allergic diseases [20–23]. We have shown in our previous studies that interference with T helper death not only skews the immune response but also determines the fate of differentiating cells [24]. Altering Th1 or Th2 death by the use of *lpr/gld* or Gr B KO mice resulted in biased and exaggerated T helper cytokine response [22, 24]. Interestingly another study where Th17 TCR AICD was compromised an exaggerated IL-17 response *albeit* at the mRNA level was evidenced [23]. Thus blocking Th1 or Th17 death and the consequent exaggerated IFN-γ or IL-17 response can significantly influencing even naïve T helper cell differentiation apart from effector T cell functions. Interestingly, several studies have also shown that Th17 cells resist AICD [19–21] and produce low level of IL-2 that is sufficient for their survival, expansion and persistence in autoimmunity [25]. Thus the significant presence of both inflammatory and pathologic Th17 cells in RA samples might point to an altered TCR AICD sensitivity apart from a skewed generation in autoimmunity [8]. Importantly mitogen activated protein kinases (MAPKs) such as Erk1/2, p38α and JNK, the TCR downstream signaling molecules known to be involved in mediating and sustaining inflammation [26–28] might alter AICD sensitivity of T helper cells and could promote their conversion to an AICD resistant inflammatory phenotype. Therefore our study was directed to understand the AICD mechanism that may allow Th17 cells existence in autoimmune RA. The most significant finding of the present study is that subtle MAPK activity controls downstream events differentially in the Th1 and Th17 helper subsets and that this subtlety allows existence of Th17 cells and additionally presents potential targets in autoimmune disorders. As proof of principle altering TCR downstream MAP Kinases signaling did alter Th17 like cells death sensitivity and thus may help target inflammatory Th1 or Th17 cells in Auto Immune diseases.

## RESULTS

### Inflammatory T helper (Th1/Th17) cells in human RA

**Figure 1:**
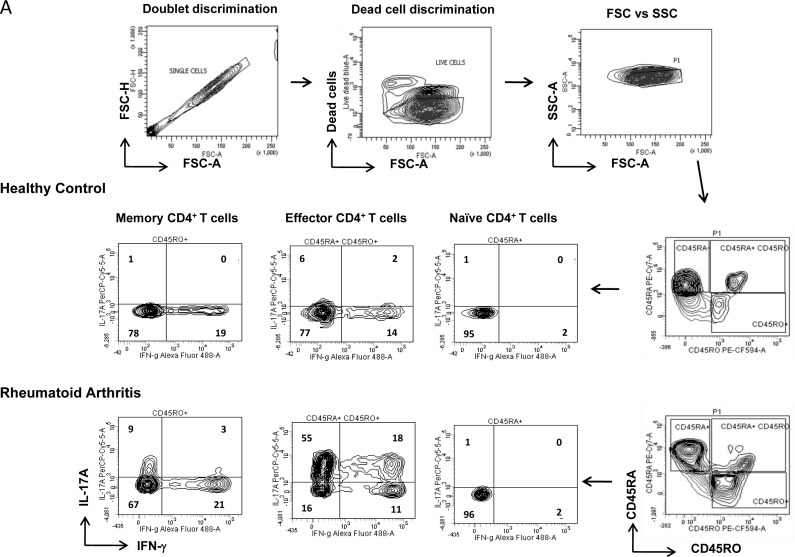

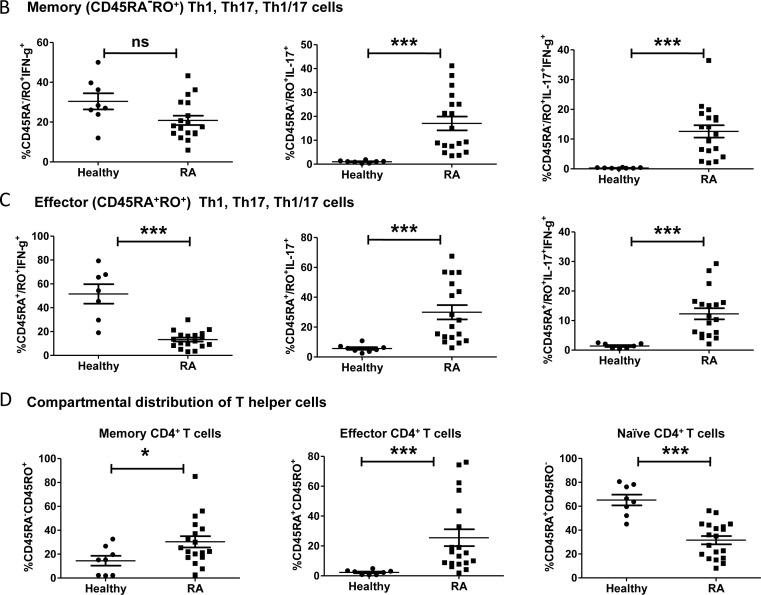
Characterization of inflammatory T helper cells in Rheumatoid Arthritis **A.** Representative gating strategy for the characterization of Th1 and Th17 cells derived from naïve, memory and effector compartments in RA and healthy control. Scatter plot with Mean ± SEM of (**D**) naïve (CD45RA^+^), effector (CD45RA^+^/RO^+^) and memory (CD45RO^+^) CD4^+^T cells; single IFN-γ^+^ or IL-17A^+^ and double positive Th1/Th17 cells derived from **B.** memory and **C.** Effector CD4 compartments from human RA (*n* = 18) and control (*n* = 9) peripheral blood samples. All samples were tested for significance by using Mann Whitney test and p value of less than 0.5 was considered as significant.

Aberrant existence of inflammatory Th1 and Th17 cells has been previously reported in RA [7, 8] but their relative contribution as effector or long-lived memory cells to inflammatory episodes and subsequent persistence is still debated. To understand their contributive roles we first examined their compartmentalized co-existence in human RA and healthy controls (Figure 1A) by flow cytometry and analyzed them by Boolean combination of gating [29, 30]. Negatively selected CD4^+^ T cells single positive for IFN-γ (Th1) or IL-17A (Th17) or dual IFN-γ/IL-17A positive (Th1/Th17) were identified by gating on CD45RA^−^RO^+^ (memory), CD45RA^+^RO^+^ (effector) and CD45RA^+^RO^−^ (naïve) populations (Figure 1A). We report the exclusive presence of significantly elevated IL-17A^+^ (Th17) and the dual IFN-γ/IL-17A^+^ (Th1/Th17) phenotypes in both the memory and effector compartments of RA patients (Figure 1B-1C). Interestingly, IFN-γ producing Th1 cells were significantly lower in effector cells and statistically insignificant in memory compartments of RA compared to control (Figure 1B-1C). To further validate T helper presence in RA we examined for their distribution as effector, memory and naïve T cells (Figure 1D). Not surprisingly while both effector and memory T helper compartments were significantly elevated, naïve T cell compartment was found to be significantly lower in RA compared to control. Further, naïve (CD45RA^+^RO^−^) CD4^+^T cells that were significantly higher in controls expressed no IFN-γ or IL-17A in both RA and control peripheral blood and thus were excluded for inflammatory T cell analyses. These results strongly suggest that inflammation in RA might maximally be contributed by the significant presence of IL-17A secreting Th17 and IFN-γ/IL-17A secreting Th1/Th17 cells in both effector and memory compartments. Our data also suggests that their exclusive presence could be indicative of an impaired T cell homeostasis operating in inflammation and auto immunity.

### Differential FasL expression in RA inflammatory T helper cells

**Figure 2:**
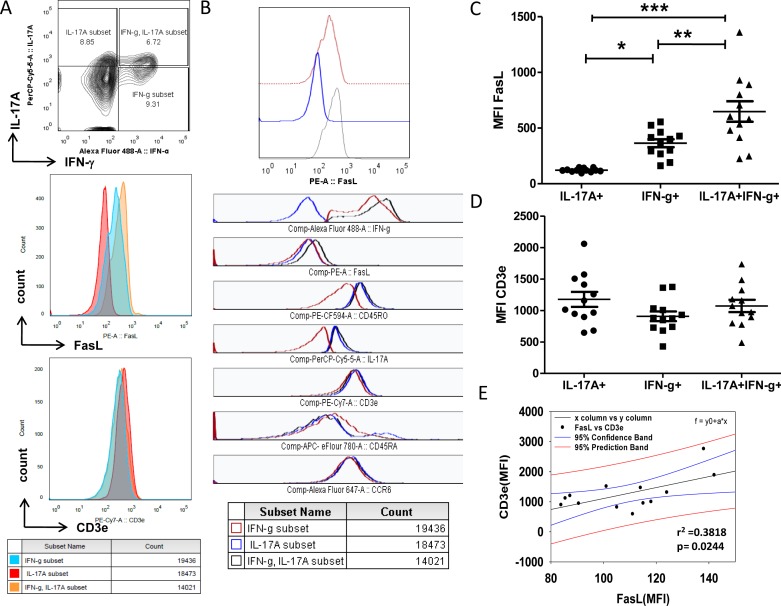
Differential expression of FasL in RA T helper cells **A.** FasL and CD3e expression in IL-17A^+^ or IFN-γ^+^ or double positive Th1/Th17 cells from human RA peripheral blood samples (*n* = 13). **B.** Differential FasL protein expression in RA T helper cells. **C.** Scatter plot with Mean ± SEM of Median Fluorescent Intensity of FasL protein in IL-17A^+^, IL-17A^+^/ IFN-γ^+^ and IFN-γ^+^ T cells of human RA blood samples (*n* = 13). **D.** Scatter plot with Mean ± SEM of CD3e protein expression in IL-17A^+^, IL-17A^+^/ IFN-γ^+^ and IFN-γ^+^ T cells of human RA blood samples (*n* = 13). Kruskal-Wallis test and Dunn's Multiple Comparison post hoc test were performed to find statistical significance using GraphPad prism and *p* < 0.5 was considered as significant. **E.** Non-linear regression analysis of FasL and CD3e proteins expression in RA CD4^+^ T cells using Sigma Plot (*r* = + 0.3818, *p* = 0.02, 95% CB) *n* = 13.

Previous studies have demonstrated that both Th1 and Th17 cells undergo AICD through the Fas-Ligand (FasL) death pathway and that AICD resistant T cells exacerbate immune pathology through cytokines [19, 20]. Thus our data from RA presenting T cells that secrete significantly higher levels of IL-17A and IFN-γ/IL-17A from both effector and memory compartments and their absence in controls suggests an impaired homeostasis that include altered TCR AICD sensitivity. Consequently to delineate the continued existence of these inflammatory Th17 and Th1/Th17 cells and to directly correlate TCR signaling intensity and TCR AICD sensitivity we studied CD3e and Fas-Ligand (FasL) expression in RA T helper cells (Figure 2A). RA Th1, Th17 and Th1/Th17 cells irrespective of their compartmental distribution were evaluated for CD3e and FasL expression by gating on IFN-γ^+^, IL-17A^+^ and IFN-γ/IL-17A^+^ populations respectively (Figure 2A-2B). FasL expression on IFN-γ^+^ Th1 and IFN-γ/IL-17A^+^ Th1/Th17 cells were significantly higher as compared to IL-17A^+^Th17 cells (Figure 2C) while CD3e expression was not (Figure 2D). In addition, IFN-γ production from Th1 and Th1/Th17 cells were found to be associated with higher FasL expression but not with IL-17A^+^ Th17 cells (Figure 2B). Thus IL-17 secreting Th17 cells inherently express lower FasL protein when compared to the other two subsets namely IFN-γ^+^ and IFN-γ/IL-17A^+^ cells. Although CD3 expression was not different in Th1 and Th17 cells, regression analysis of FasL and CD3e expression on total CD4 cells (sum of all subsets) surprisingly shows significant positive correlation (Figure 2E). Thus similar CD3e expression and varied levels of FasL protein suggest events downstream TCR signaling including differential MAPK activity as seminal in dictating FasL expression in these cells. Additionally these results suggest that differential FasL expression from Th17 and Th1/Th17 cells could affect their differential TCR AICD sensitivity and might influence their inflammatory and or pathological existence.

### Low FasL expression on RA Memory Th17 cells

**Figure 3:**
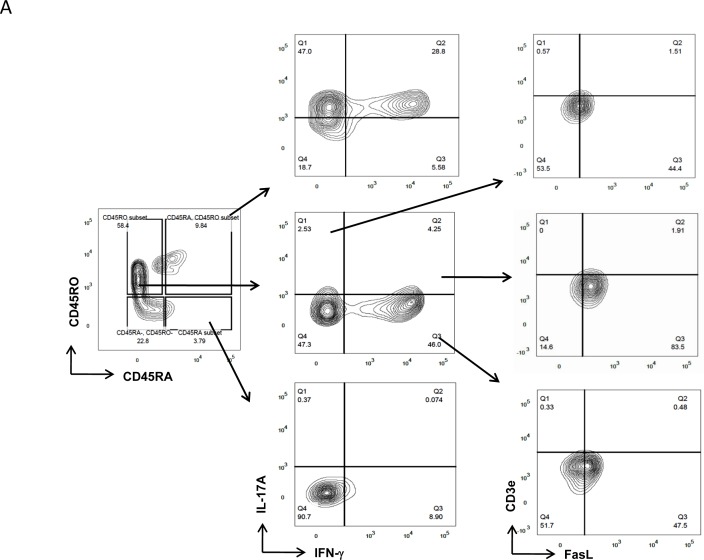

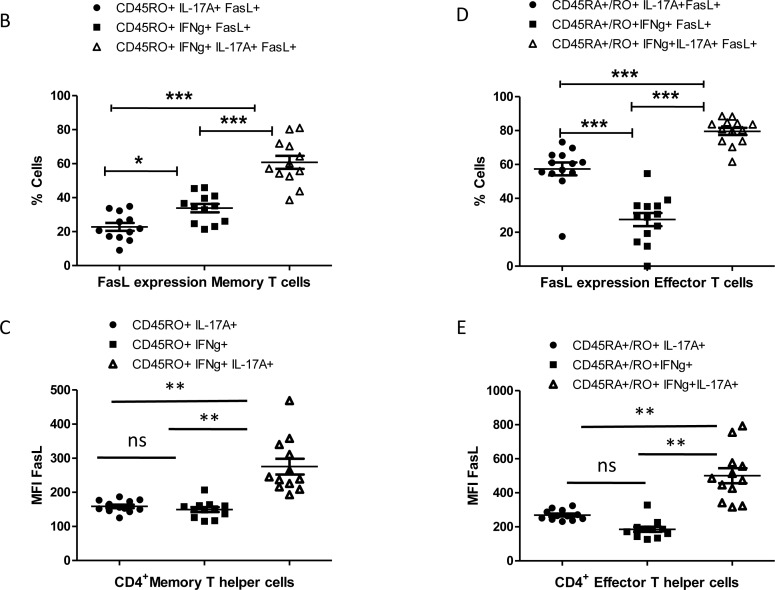
FasL expression in Th1 and Th17 cells of memory and effector compartment in RA **A.** Representative gating strategy for the characterization of FasL expressions in Th1 and Th17 cells derived from naïve, memory and effector compartments of RA samples (*n* = 13). **B.** Scatter plot with Mean ± SEM of % FasL^+^ and **C.** Median Fluorescent Intensity of FasL in population of IL-17A^+^ or IFN-γ^+^ and double positive Th1/Th17 cells of memory CD4 T compartments in RA. **D.** Scatter plot with Mean ± SEM of % FasL^+^ and **E.** Median Fluorescent Intensity of FasL in population of IL-17A^+^ or IFN-γ^+^ and double positive Th1/Th17 cells of Effector CD4 T compartments in RA. Data was tested for significance by using one-way ANOVA and Tukey's Multiple Comparison post hoc test and *p* < 0.5 considered as significant.

The above results show differential FasL expression in IL-17A producers and could suggest an inherent difference in the TCR AICD sensitivities of Th17 and Th1/Th17 cells. To ascertain and identify the cause for both inflammatory and pathogenic Th17 cell presence in RA, we then examined for FasL expression in RA IFN-γ^+^(Th1), IL-17A^+^(Th17) and IFN-γ/IL-17A^+^(Th1/Th17) cells from long-lived memory or effector compartments (Figure 3A). To our surprise memory and effector IFN-γ/IL-17A^+^ cells were more percent positive for FasL protein than IFN-γ^+^ and IL-17A^+^ cells (Figure 3B and 3D). Additionally memory and effector dual IFN-γ/IL-17A^+^ cells expressed significantly higher levels of FasL while memory and effector single positive IFN-γ^+^ and or IL-17A^+^ cells expressed low levels of FasL (Figure 3C and 3D). Thus both at the cytokine compartment level and at the functional compartmental level IFN-γ/IL-17A^+^ cells were significantly higher FasL expressors than single IFN-γ^+^ or IL-17A^+^ cells. Interestingly, the level of FasL expression observed in both effector and memory IFN-γ^+^ and IL-17A^+^ cells were similar in RA (Figure 3C and 3D). Although increased FasL positive effector Th17 cells were encountered but their FasL expression levels were similar to effector Th1 cells. These results suggest that the effector and memory dual IFN-γ/IL-17A^+^ “Th1/Th17 subset” might be susceptible to AICD but effector IFN-γ^+^ and memory IL-17A^+^ could be insensitive to AICD due to their low FasL expression in RA. Here we assume that this might be a result of the highly skewed double positive cells, as effector IFN-γ^+^ cells are known to generate higher FasL protein. Interestingly IL-17A^+^FasL^+^ cells, the least in the memory compartment also expressed significantly lower FasL protein (Figure 3B and 3C). These results also highlight an inherent capability for long-lived memory IL-17A^+^ cells to escape AICD through low FasL expression.

### RA T helper cells are resistant to activation induced cell death

**Figure 4:**
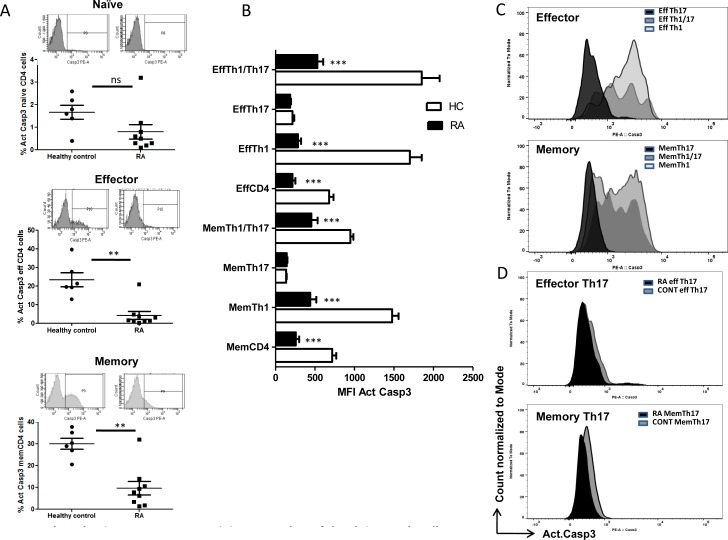
Active Caspase-3 as an index of AICD in Human RA and control T helper cells **A.** Scatter plot with Mean ± SEM of % Active Caspase3 and representative flow histogram of compartmental T cells from human RA and control. **B.** Mean ± SEM of MFI Active caspase3 in compartmental T helper cells from human RA (*n* = 9) and control (*n* = 6). **C.** A representative histogram overlay of Active caspase3 in Effector (upper) and Memory (lower) Th1, Th17 and Th1/Th17 cells from human control. **D.** A representative histogram overlay of Active caspase3 in Effector Th17 (upper) and Memory 17 (lower) cells from human RA (*n* = 9) and control (*n* = 6). Two-way ANOVA and Bonferroni posttests were performed to find statistical significance using GraphPad prism and *p* < 0.5 was considered as significant.

Our analyses of T helper compartments suggested a differential FasL expression between effector and memory T helper cells in RA. We further examined for executor caspase3 activity in RA and control T helper cells as a direct correlate to understand their TCR AICD sensitivity. Both effector and memory RA CD4^+^T cells were observed to have significantly higher active Caspase-3 than control (Figure 4A). Dependably RA Th1 and Th1/Th17 from both effector and memory compartments were found to show significantly low active Caspase3 than their counterparts in control (Figure 4B). Further Th1/Th17 cells that express high FasL were found to have lower active caspase3 in RA compared to control, pointing to lowered AICD sensitivity than Th1 cells. Although Th1/Th17 cells are designated as “pathogenic Th17 cells”, their absolute contribution as causative or in response to RA is unclear. We assume that Th1/Th17 cells may be generated from IL-17A^+^Th17 cells in response to RA inflammation and additionally these AICD resistant cells may be fratricidal because of high FasL expression and might indicate initiation of negative feedback of Th17 through IFN-γ. However among the T helper cells, Th17 cells from the Effector and Memory compartments were observed to show significantly lower caspase-3 activities in both control and RA (Figure 4B-4D). Detailed analysis of active caspase-3 (Supple Figure 1) revealed Th17 cells' inherent ability to resist AICD as evinced by the lowered active caspase-3 activities in both control and RA subjects. These results strongly suggest that RA Th1 and Th1/Th17 cells could possibly develop AICD resistance as a consequence of RA inflammation and that Th17 cells' inherent AICD resistance could exacerbate inflammation in RA.

### *In vitro* generated Th17 cells are resistant to activation induced cell death

Our results demonstrate low FasL protein and active caspase-3 in IL-17A^+^ (Th17) cells in the memory compartment and thus this inverse co relation needed further elaboration. Despite stringent exclusion criteria for “control” humans the absence of even minimal infections, varied immune status, dietary habits, etc. compound our interpretation of human peripheral blood derived T helper subsets. Thus to examine for FasL profile in T helper cells and to compare their AICD susceptibility, we first generated murine T helper subsets *in vitro* and characterized them for their signature cytokines and transcription factors (Supple Figure 2A-C). Post differentiation murine Th1 cells exhibit dual positivity for IFN-g and T-bet while Th17 cells were dual positive for IL-17 and ROR-γ (Supple Figure 2A-C). Importantly these highly specific T helper subset markers and cytokine analyses conferred reliability on the differentiated T helper subset and allowed mechanistic dissection of their death pathways.

**Figure 5:**
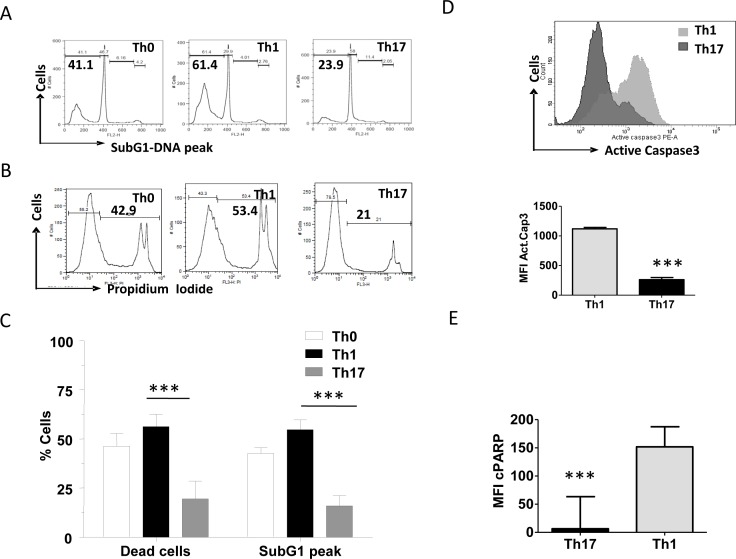
Characterization of differential AICD susceptibility in T helper cells **A.** Representative histogram of Sub G1 peaks (upper) and **B.** loss of viability (lower) in Th0, Th1 and Th17 cells after 16 hrs of anti-CD3 induced reactivation. **C.** Mean and SEM of percent SubG1 DNA and PI^+^ dead cells of T helper cells. **D.** Mean and SEM of Active Caspase3 (below) and representative histogram (above) of active Caspase3 during AICD of Th1 and Th17 cells. **E.** Mean and SEM of cleaved PARP during TCR-AICD of Th1 and Th17 cells. Data represent at least three independent experiments. Data was tested for significance by using one way ANOVA and Tukey's Multiple Comparison post hoc test and *p* < 0.5 considered as significant.

Differentiated Th0, Th1 and Th17 cells died on TCR AICD induction with anti-CD3 but with different kinetics (Figure 5A-5C). Apoptosis and cell death were studied by four different parameters such as DNA fragmentation (SubG1 peak), compromised plasma membrane permeability (propidium iodide positive dead cells), active Caspase-3 and Poly (ADP-ribose) polymerase cleavage (c-PARP). Consistent with previous reports [19, 21, 31] we observed that Th17 cells underwent significantly lower cell death compared to Th0 and Th1 cells (Figure 5A-5E). Our studies confirm that T helper subsets die through TCR AICD post differentiation and the three subsets mentioned utilize the canonical caspase death pathway to die and amongst the tested T helper subsets, Th17 was the least susceptible to TCR induced death.

### Death sensitive and resistant T helper subsets exhibit differential MAP kinase activities

**Figure 6:**
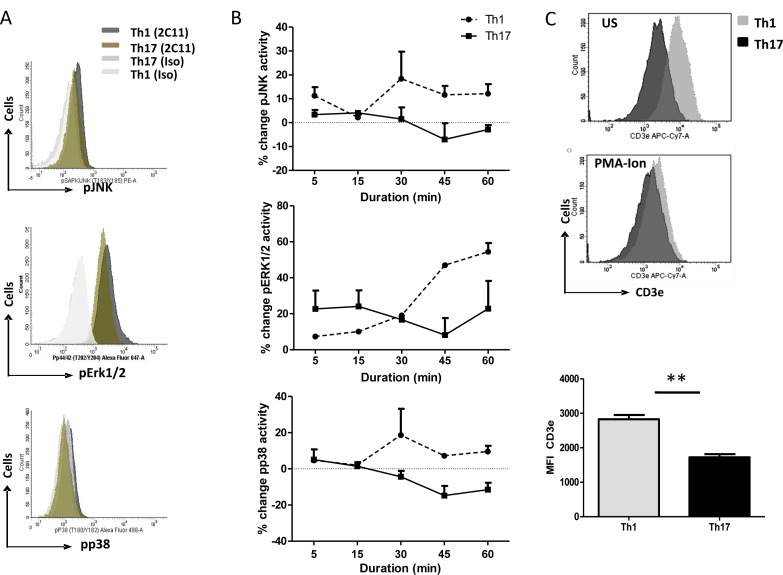
Differential MAPK signaling in T helper cells **A.** Representative flow cytometry histogram of phospho-JNK (upper), phospho-ErK1/2 (middle) phospho-p38 (lower) in Th1 and Th17 cells after 30min of anti-CD3 (2C11) reactivation by secondary cross-linking with anti-Hamster IgG. **B.** Time kinetics of MAPK activity in Th1 and Th17 cells after anti-CD3 (2C11) reactivation by secondary cross-linking with anti-Hamster IgG, phospho-JNK (upper), phospho-ErK1/2 (middle) phospho-p38 (lower). **C.** CD3e expression in Th1 and Th17 cells of Balb/c or C57BL-6 mice with or without PMA-Ionomycin stimulation.

To elucidate the role of TCR signaling in AICD resistance of Th17 cells, we examined MAPK activity downstream TCR activation in Th1 and Th17 cells (Figure 6A). Time kinetic studies on Th1 and Th17 cells revealed that all three MAP kinases namely JNK, p38 and Erk1/2 activities were mostly lower in Th17 cells compared to Th1 (Figure 6B). Further we performed western blot analyses of MAPKs in Th0, Th1 and Th17 cells with or without antiCD3e stimulation. Phospho-p38, p42 and p44 levels were higher with CD3e stimulation compared with unstimulated Th0 and Th1 (Supple Figure 3). However these kinases were lowered in CD3e stimulated Th17 cells compared with unstimulated Th17 cells (Supple Figure 3). These data suggest that Th17 cells had low MAPK activity under CD3e stimulation. Thus antiCD3e stimulation of Th0, Th1 and Th17 cells exhibited low MAPK activities only in Th17 cells and suggests that upstream TCR-signaling events could dictate this differential phosphorylation. To examine for differential MAPK activity we further examined for CD3e expression on Th1 and Th17 cells. Surprisingly CD3e expression on unstimulated and PMA-Ionomycin stimulated Th1 cells were significantly higher when compared to similarly treated Th17 cells (Figure 6C). These results are contra indicated in human RA Th1 and Th17 cells where CD3e expression on human Th1 and Th17 cells were not different (Figure 2D), however, CD3e expression on murine Th1 cells were consistently higher across the strains (Figure 6C). These results suggest that *ex vivo* differentiated murine Th1 and Th17 cell CD3e expression may be reflective of very early rounds of activation unlike RA Th1 and Th17. In addition RA CD3e expression was analyzed exclusively on IFN-γ (Th1) or IL-17A (Th17) or dual IFN-γ/IL-17A positive (Th1/Th17) (Figure 2B and 2D) and maybe reflective of the inflammatory T cell phenotype. The differential or low MAPK activity in Th17 cells maybe a direct co relate of CD3e expression and thus the subsequent lowered downstream MAPK activity may alter T helper TCR AICD.

### MAP kinase activities modulate T cell apoptosis

**Figure 7:**
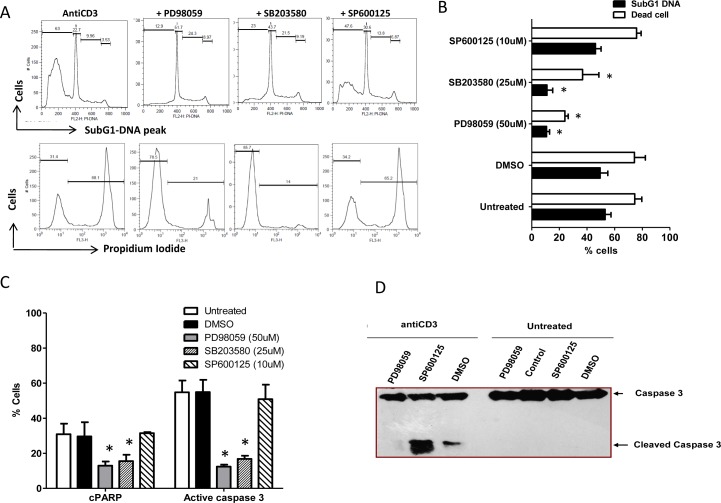
T helper cells AICD and MAPK **A.** Representative histogram of Sub G1 peaks (upper) and loss of viability (lower) in Th1 like A1.1 cells treated with or without MAPK inhibitors: PD98059 (50uM), SB203580 (25uM) and SP600125 (10uM) and after 16 hours (overnight) of anti-CD3 induced reactivation. **B.** Percent SubG1 DNA and PI^+^ dead cells are represented as Mean and SEM. **C.** Mean and SEM of Active Caspase3 and cleaved PARP in Th1 like A1.1 cells treated with or without MAPK inhibitors such as PD98059 (50uM) for Erk1/2, SB203580 (25uM) for p38 and SP600125 (10uM) for JNK and after 16 hrs (overnight) of anti-CD3 induced reactivation. **D.** Western blot analysis of Activate Caspase3 in similarly treated A1.1 cells. Data represent at least three independent experiments and was tested for significance by using one-way ANOVA and Tukey's Multiple Comparison post hoc test and *p* < 0.5 considered as significant.

AICD resistant Th17 cells exhibit lower MAPK activity upon CD3e stimulation. To corroborate the significance of low MAPK activity and AICD resistance in these cells, we modulated MAPK activities by the use of specific inhibitors on AICD sensitive Th1 like A1.1 cells. We assessed cell death by four different parameters such as SubG1 peak, propidium iodide positivity, active Caspase-3 and PARP cleavage in these cells. Erk1/2 and p38 Inhibition was observed to protect A1.1 against TCR induced cell death as assessed by significantly low subG1-peak, dead cells, active caspase3 and c-PARP compared to JNK inhibited cells (Figure 7A-7C). Interestingly Erk inhibited T helper cells upon anti-CD3 stimulation showed no cleaved caspase-3 fragments compared to similarly treated JNK inhibited and mock cells (Figure 7D). These results together strongly suggest that the low p38 and Erk1/2 activities post TCR activation could lead to decreased AICD.

### Erk1/2 and p38 controls FasL protein expression and degranulation in T helper cells

**Figure 8:**
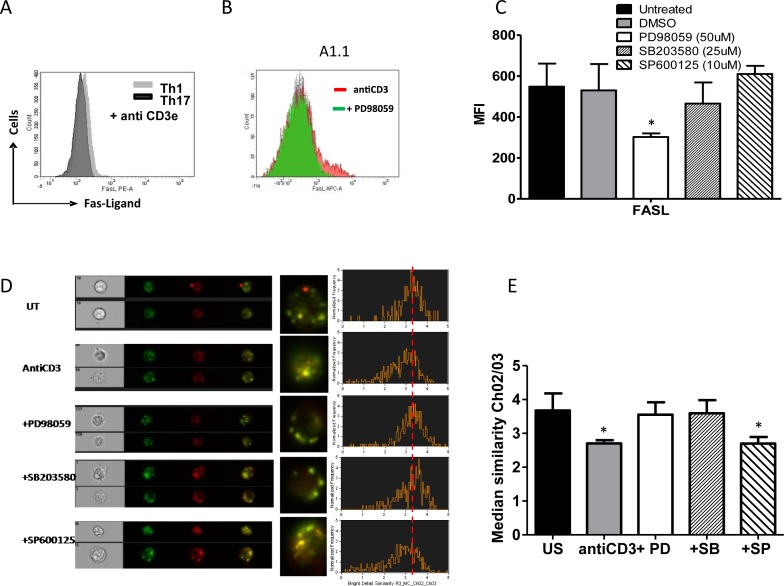

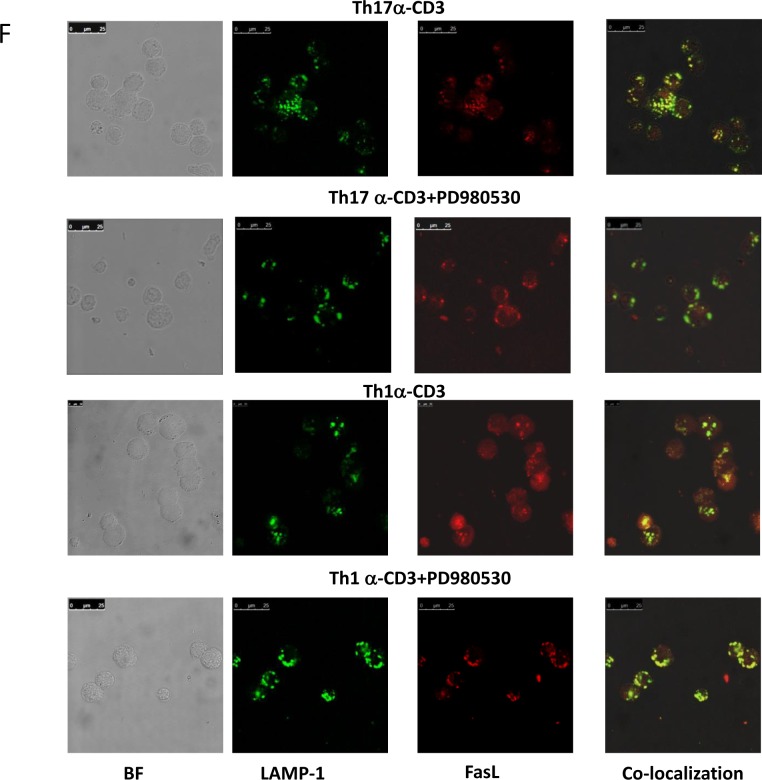
MAPK on FasL protein expression and degranulation in T helper cells **A.** Representative histogram overlay of FasL expression in Th1 (grey) and Th17 cells (black) after anti-CD3 stimulation for 10 hrs. **B.** Representative histogram overlay of FasL expression in A1.1 cells treated with antiCD3 alone (red) and antiCD3 + PD98059 (green). **C.** Mean and SEM of Total FasL expression (MFI) in Th1 like A1.1 cells treated with and without MAPK inhibitors such as PD98059 (50uM) for Erk1/2, SB203580 (25uM) for p38 and SP600125 (10uM) for JNK and after 10 hrs of anti-CD3 induced reactivation. **D.** A representative image and histogram of Imaging flow cytometry of FasL protein degranulation from lysosomal co-localization with LAMP-1 FITC (green) and FasL PE (red) in A1.1 cells treated with and without MAPK inhibitors such as PD98059 (50uM) SB203580 (25uM) and SP600125 (10uM) and after 10 hrs of anti-CD3 induced reactivation. **E.** Mean and SEM of median similarity of Ch02 (LAMP-1-FITC) and Ch03 (FasL PE) in similarly treated A1.1 cells. **F.** Representative confocal image of FasL protein degranulation from lysosomal co-localization with LAMP-1 FITC (green) and FasL PE (red) in Th1 and Th17 cells.

MAPK's activities specifically Erk and p38 affect Th1 and Th17 death and to further understand and consolidate their mechanistic roles we examined for Fas, FasL and TRAIL protein expression and FasL degranulation upon anti-CD3 stimulation (Figure 8A-8F and supplement Fig 4A-C). Th1 (Figure 8A) and A1.1 cells (Figure 8B) were found to exhibit high FasL protein on TCR stimulation but Th17 cells (Figure 8A) and Erk inhibited A1.1 cells (Figure 8B) expressed significantly lower FasL protein (Figure 8A and 8B). Total FasL expression was unaffected by p38 or JNK inhibition (Figure 8C) and there was no significant difference in the expression of Fas or TRAIL (supple Figure 4B and C). These data suggest that Erk activity regulates FasL expression but did not delineate p38's anti apoptotic role. Since p38 did not affect total FasL expression but rescued both Th1 and Th17 cells we then examined for FasL degranulation (Figure 8D and 8E). Imaging flow cytometry and confocal microscopy analysis of FasL degranulation based on lysosomal co localization and release of synthesized FasL protein using CD107a (LAMP-1FITC) and FasL PE (CD178) co localization with median similarity index as a measure of co localization revealed a unique role for p38. Anti CD3 stimulated degranulation of FasL significantly decreased by decreased similarity index indicating that p38 activity controlled FasL degranulation from the lysosome. Interestingly FasL degranulation was significantly high in Th1, A1.1 and JNK inhibited A1.1 cells while Th17, Erk inhibited Th1 and p38 or Erk inhibited A1.1 cells showed lowered FasL degranulation (Figure 8D-8F). Together our results prove that Erk and p38 affect T helper death by affecting FasL protein expression and its subsequent degranulation. Importantly FasL protein expression and secretion under normal physiological conditions were always lower in Th17 cells than Th1 cells due to lowered Erk1/2 and p38 activity and could very significantly contribute to AICD resistance of Th17 cells.

### Erk2 mediates TCR induced cell death in T helper cells

**Figure 9:**
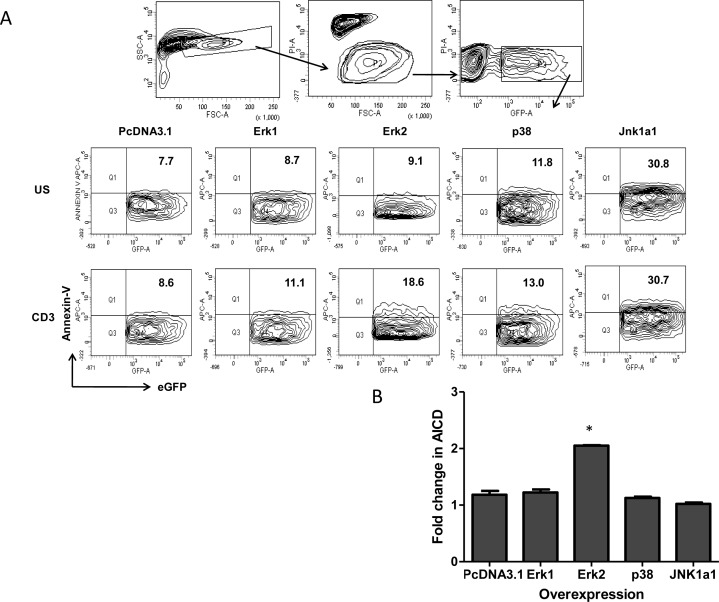
Over expression of MAPK in T helper cells AICD **A.** A representative gating strategy to study AICD (Annexin-V binding) in transiently transfected Th17-like EL4 cells with Wild type MAPK such as Erk1, Erk2, p38 and Jnk1a1 plasmids. **B.** Mean and SEM of fold increase (Annexin-V binding) AICD in MAPK over expressed Th17 like EL4 cells. Data was tested for significance by using one-way ANOVA and Tukey's Multiple Comparison post hoc test and *p* < 0.5 considered as significant.

Our experiments show that low Erk and p38 activity reduce FasL expression and degranulation in both Th1 and Th17 cells directly impacting TCR-AICD. Consequently the inherent low p38 and Erk activity encountered in physiologically differentiated Th17 *versus* Th1 cells strongly suggest that Th17 cells' in-built AICD resistance could be altered by modifying MAPK activity. Thus Th17 like EL4 cells were examined for the reverse consequence of higher MAPK activity wherein we evaluated their AICD post over expression of wild type Erk1, Erk2, p38 and Jnk1a1 MAPK's (Figure 9A and 9B). Amongst the MAPK's we over expressed, JNK1a1 over expression as such caused high Annexin-V binding and TCR stimulation of these cells did not alter their death profile suggesting that JNK1a1 might not influence TCR AICD. Erk1 and p38 activity over expression did not affect background death and also did not significantly enhance TCR AICD of EL4 cells. A two-fold increase in Annexin V^+^ cells in Erk2 over expressed and TCR stimulated cells (Figure 9B) strongly suggested that Erk2 is critical for TCR AICD. From our previous results with primary Th1 and Th17 cells, this data suggests that Erk2 activity could determine death sensitivity in EL4 cells by enhancing FasL protein synthesis while the combination of FasL synthesis and de granulation could determine primary T helper cells death. Thus altering Erk2 activity in Th17 predominant inflammatory disease and subsequent FasL synthesis and de granulation could directly influence inflammation and immune pathology.

## DISCUSSION

Inflammation driven T helper 17 generation and/or Th17 cellular resistance to homeostatic deletion are critical in determining Th17 persistence in Rheumatoid Arthritis. We have shown in our previous studies that interference with T helper death not only skews the immune response but also determines the fate of differentiating cells. Altering Th1 or Th2 death by the use of *lpr/gld* or Gr B KO mice resulted in biased and exaggerated T helper cytokine response [22, 24]. Interestingly inhibition of Th17 death also results in an exaggerated IL-17 response albeit at the mRNA level [23]. Thus blocking Th1 or Th17 death will result in an exaggerated IFN-γ or IL-17 response significantly influencing even naïve T helper cell differentiation apart from effector cell function. The present study attempted to understand the AICD mechanism that favors Th17 existence in Rheumatoid Arthritis. Our results strongly suggests that Inherent low Erk and p38 activities, reduced Fas Ligand expression and degranulation and lowered active caspase-3 in Th17 cell lead to their impaired AICD and may crucially influence their subsequent aberrant existence in RA. Thus TCR activated events including MAPK's; death ligands and terminal caspase activity dictate the balance between immune tolerance and inflammation mediated by Th17 cells. Amongst T helper subsets that we examined, Th17 cells were the most resistant subset to anti-CD3 induced TCR AICD. Importantly our study suggests that very early and late TCR events including CD3 levels, Erk and p38 activities, Fas and FasL protein, caspase activities etc. are the determinants of Th17 TCR AICD. Two critical proteins CD3e and FasL that exhibit differential profiles in Th1 and Th17 cells impact TCR signaling strength and the subsequent MAPK activity exquisitely determines TCR AICD. Amongst MAPKS' Erk activity is critical in determining Th17 AICD insensitivity [32, 33] and may potentially serve as a therapeutic target.

MAPK activity especially Erk is critical in determining naïve T helper cell homeostasis where sustained low Erk activity rescues T cells from death during thymic positive selection while robust rapid Erk activity kills cells via AICD during thymic negative selection [34]. Post negative selection Erk critically determines terminal differentiation of T helper subsets and once terminally differentiated these T helper subsets also display distinct Erk profiles. Subsequently the slightly higher constitutive levels of Erk phosphorylation in primary Th17 cells could protect them against basal and background death [35] that are generally encountered in high inflammatory environment and could unduly favor Th17 propagation. It has to be noted that basal Erk activity might be a consequence of the different driving cytokines during Th17 and Th1 differentiation. However the biological consequence and relevance of lowered Erk activity upon TCR reactivation leading to lowered FasL protein and reduced caspase activity in Th17 cells suggest direct proof of principle for their AICD resistance and higher incidence in inflammatory diseases including psoriasis, RA, inflammatory bowel disease and multiple sclerosis [36–39]. Evidence from other groups and ours (unpublished data) suggest that lowered Erk activity post TCR activation tends to favor Th17 propagation [40, 41] and Erk2 enhancement sensitizing Th17 cells to TCR AICD has major consequence attached.

Altered AICD sensitivity between Th1 and Th17 cells as a consequence of differential MAPK activity and low FasL expression suggest their direct implication in autoimmune diseases and we extrapolated these findings in Th17 mediated RA immuno pathology. Although recent studies have shown a resurgence of Th1 cells in RA synovial fluid [3, 7, 8, 12] Th17 cells are the most prominent T cell type in human RA synovial tissue and peripheral blood. Our findings strengthens the Th17 angle through the exclusive presence of inflammatory IL-17A^+^ (Th17) and dual IFN-γ^+^/IL-17A^+^ (Th1/Th17) cells but significantly low IFN-γ^+^(Th1) in RA peripheral blood. Two seminal points that favor Th17 cell in RA inflammation from our studies are that: Th17 cells are known to be osteoclastogenic capable of accelerating bone destruction in RA and secondly Th17 cells are highly resistant to TCR stimulation induced cell death [20]. Extrapolating our murine primary and human RA studies we infer that the pathogenic and inflammatory Th17 cells are the IL-17A^+^ (Th17) and dual IFN-γ^+^/IL-17A^+^ (Th1/Th17) cells that might persist due to their inherent AICD insensitivity and contribute to the episodic inflammation [42]. However it is too simplistic to assume that lowered Erk activity, FasL and caspase activity alone determine Th17 presence in RA, as these might also present in “controls”. The importance of the inflammatory cytokine milieu altering Th17 phenotype especially their inflammatory cytokine profile and their AICD sensitivity has to be considered. Also the presence of effector “pathogenic Th17 cells” dual IFN-γ^+^/IL-17A^+^ (Th1/Th17) in peripheral blood suggests “plasticity” that could have been driven by the inflammatory cytokine milieu [43, 44] that is generally not encountered in *in-vitro* generated primary T helper cells. The single most important finding in our human inflammatory disease studies is the absence of dual Th1/Th17 cells in “controls” suggesting that autoimmunity is associated with the presence of persistent inflammatory Th17 and Th1/Th17 cells. In addition the presence of such inflammatory phenotypes raises the issue if the dual phenotypes, “pathogenic Th17 cells” are the cause or effect of RA inflammation.

TCR AICD of both murine and human T-cell subsets such as Th1 and Th17 cells is mediated by Fas and differentially regulated by Fas-ligand [45, 46]. Interestingly though the differentiation of Th17 and Th1 cells is driven by different cytokines they die through the common Fas-FasL pathway. Moreover Th17 cells differentiated in the absence of TGF-β also undergo FasL dependent TCR AICD and bears commonality with Th1 and classical Th17 cells. Hence FasL mediated canonical death pathway seems to be the preset TCR AICD pathway for both physiological and pathological Th1 and Th17. Apart from differential FasL expression encountered in Th1, Th17 and dual Th1/Th17 cells from both effector and memory compartments of RA, there is low AICD in Th1 and Th1/Th17 cells in RA as compared to their counterparts in control. Although RA Th1 cells resist AICD but their actual contribution to RA can be minimal due to their low occurrence. Hence AICD resistance of Th1/Th17 and Th17 cells' significant presence in RA strongly suggests their potential pathological roles in RA.

Interestingly, in possibly the most emphatic regulatory role for Th1 to the development and maintenance of RA is the autoimmune murine arthritis model in IFN-γ receptor-deficient mice. These mice exhibit severe symptoms of arthritis due to Th17 dominance [47] and the complete absence of Th1 cells suggest a counter regulatory mechanism by Th1 cells. These findings suggest the importance of Th1 cells and may suggest a control mechanism in human RA.

Mechanistic understanding of Th1 and Th17 MAPK pathways emphasis their role in activation, differentiation and their final termination. The seminal finding that enhanced Erk activity could sensitize death resistant Th17- like EL4 cells strengthen the importance of Erk2 activity and establish for the first time Erk2 as a death sensitizer in T helper subsets. In conclusion our results strongly suggest that Erk1/2 and p38 activities are important for TCR mediated FasL protein up regulation (include their expression and degranulation) and T helper cells AICD sensitivity. Thus Erk2 and p38 could be promising targets in autoimmune diseases to remove the aberrant or pathological T helper subsets.

## MATERIALS AND METHODS

### Human and mice

The present study was approved by Institutional Human Ethical Committee and permitted to collect blood samples from human healthy volunteers and Rheumatoid arthritis patients. Recruitment of healthy subjects was based on inclusion criteria and RA subjects included in the study were based on the ACR/EULAR criteria [1], clinical history and rheumatoid factor positivity. Samples were collected from control and RA subjects with their informed consent. Demographic data of control and RA volunteers are depicted in Supple Table. 1.

**Figure 10:**
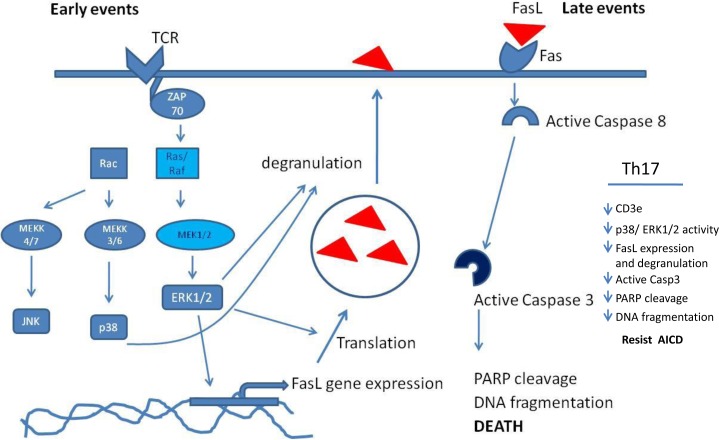
MAP kinase mechanism in T helper AICD A schematic figure that represent the MAP Kinase involvement in AICD

Wild type strain of Balb/c and C57BL/6 mice were obtained from National Institute of Immunology (New Delhi, India) or from Imgenex (Bhubaneswar, India) and maintained in *12hrs-Dark-light cycle* in a pathogen free animal facility at Institute of Life Sciences, Bhubaneswar, with food and water provided *ad libitum.* Institutional Animal Ethical Committee approved the present study and age and sex matched inbred wild type Balb/c and C57BL/6 mice were used.

### Antibodies and reagents

Dynal Mouse CD4^+^ T Cell enrichment kits for negative selection were purchased from Invitrogen, California, USA. Anti-T-bet conjugated with PerCP-Cy5.5, anti-ROR-γ conjugated with APC or PE, anti-GATA-3 conjugated with PE, anti human IFN- g conjugated with Alexa Flour 488, anti human IL-17A PerCP-cy5.5, (eBioscience, San Diego, CA, USA). Antibodies against mouse IFN-g conjugated with PE-cy7, IL-17A PerCP-cy5.5, FasL PE or APC, LAMP-1 FITC, FAS PE-cy7, TRAIL PE, CD4 FITC, CD8a PerCP, CD62L PE, CD69 PE-cy7, CD3e APC-cy7, Active Caspase-3 PE, Cleaved PARP FITC and Antibodies against human CD4 PE, CD8 FITC, CD69 APC, CD45RO CF594, CD45RA PE-Cy7, CCR6 Alexa Flour 647, anti phospho p38 PerCP-cy5.5 and Cytofix/cytoperm wash buffers were purchased from BD Biosciences (San Jose, CA, USA) and Foxp3 fixation- permeabilization buffer kits were obtained from eBioscience (San Diego, CA, USA). Purified antibodies against CD3e (145-2C11), CD28 (37.51), IFN-g (XMG1.2) and IL-4 (11B11) were purchase from Bio X Cell, Inc (West Lebanon, USA) and eBioscience (San Diego, CA, USA). Recombinant Human TGF-β 1 and recombinant mouse IL-6 were from ProSpec (New Jersey, USA), recombinant mouse IL-12p70 and recombinant human IL-2 (eBioscience San Diego, CA, USA). Anti phospho antibodies against Erk1/2 Alexa Flour 647, JNK PE, p38 Alexa Flour 488, purified western blot antibodies against BcLxL, phospho Erk1/2, phospho p38, phospho JNK and biotin conjugated anti-phopho Erk1/2 were purchased from Cell signaling Technology Inc (Danvers, MA USA), anti-c-FLIP and anti- BIM (eBioscience San Diego, CA, USA) and β-actin, GAPDH and HRP conjugated secondary antibodies from Santa Cruz Biotechnology (Texas, USA). 2D quant kit (GE, health care Life Sciences, USA) and Bradford reagent (Bio-Rad, USA) were used for protein quantification. Brefeldin A and all other chemicals were purchased from Sigma Aldrich -Corporation (St. Louis, MO, USA). Plasmids such as Erk1, Erk2, JNK1a1 and p38α for over expression studies were purchased from Addgene repository (Cambridge, MA USA). The enhanced GFP plasmid was from Clontech Laboratories (CA, USA).

### Murine CD4^+^T cell isolation and differentiation

Naïve Mouse CD4+ T cells were purified from spleens and lymph nodes of Balb/c or C57BL/6 mice. These cells were then cultured in 6 well plates pre coated with 1 μg/ml anti-CD3 (clone 145-2C11) and 2 μg/ml anti-CD28 mAbs (clone 37.51) in complete RPMI medium (RPMI 1640 (# P04-16500, PAN-Biotech GmbH, Germany) supplemented with 10% FBS Australian origin (#1302-P100402, PAN-Biotech GmbH, Germany), 50 μM 2-ME, 100 U/ml penicillin and 100 μg/ml streptomycin (# P4333-100mL, Sigma-Aldrich, USA) in various polarizing conditions as mentioned. Th17 differentiation condition included addition of exogenous IL-6 (40 ng/ml), TGF-β1 (1.5 ng/ml), anti- IFN-g (10 μg/ml, clone XMG 1.2) and anti-IL-4 (10 μg/ml, clone 11B11) and cultured for 4 days; Th1 included addition of exogenous rhuIL-2 (100 U/ml), IL-12 (10 ng/ml) and anti-IL-4 (10 μg/ml, clone 11B11) while Th0 required only rhuIL-2 (100 U/ml) and were cultured for 3 days. For resting, cells were washed off anti-CD3/CD28 and transferred to another plate, supplemented only with their respective polarizing cytokines and anti-cytokine antibodies. On 5^th^ day from the start of culture, cells were again washed and characterized for complete differentiation.

### Human T helper cell stimulation and characterization

CD4^+^ T cells were purified from PBMC of Human peripheral blood by negative selection using column free Dynal beads as per manufacturer's protocol (Invitrogen, California, USA). They were stimulated immediately with PMA (5ng/mL) - Ionomycin (1ug/mL) for 12 hours in RPMI-1640 complete medium (RPMI 1640 supplemented with 10% FBS Australian origin, 50 μM 2-ME, 100 U/ml penicillin and 100 μg/ml streptomycin). Brefeldin A (10ug/mL) and Monensin (2 uM) were added after 4 hours of stimulation in the culture for Intracellular protein staining.

### Characterization of T helper cell AICD

Fully differentiated T helper cells were reactivated with anti-CD3 (1ug/ml plate bound) for about 16 hours under standard cell culture condition at a density of 1 × 10^6^ cells per ml in complete medium to study AICD. The cells were stained with propidium Iodide in presence of triton X-100 for detection of DNA fragmentation (subG1) or with propidium Iodide without triton X-100 for the identification of loss of plasma membrane permeability. Similarly treated cells were also stained for Active Caspase-3 and Cleaved PARP after fixation and permeabilization as mentioned elsewhere. Surface expression of FasL, TRAIL and FAS were analyzed by flow cytometry in T helper cells treated with plate bound anti-CD3 for 10 hours. Total FasL expression was studied in fixed and permeabilized cells with/without Brefeldin A blocking during reactivation.

Human RA and control T helper cells were stimulated with PMA-Ionomycin for about 14-16hrs and evaluated for active caspase3 using Flow cytometry.

### Characterization of T helper cells phenotype by flow cytometry

Intracellular cytokine staining for IL-17A, IFN-γ and other cytokines were performed by as per standard operating protocol [48]. Briefly, cells were stimulated with PMA/Ion or antiCD3e and cytokine secretion was blocked with Brefeldin A (10 μg/ml) after 2 h of stimulation. Cells were washed; Fc blocked with 2% FCS PBS and then stained with conjugated CCR6, CD45RA, and CD45RO antibodies for 30 minutes. After washing cells were fixed with BD fix perm solution for 20 min at RT, washed again in perm/wash buffer and re-suspended in 50 μl of BD perm/wash buffer for intracellular staining with anti IL-17A and IFN-γ. The stained cells were acquired on a LSR Fortessa (BD, San Diego, CA) and analyzed by FlowJo or FACS DIVA 6 software. Isotype controls were used to check background fluorescence. For the measurement of TF's, cells were fixed and permeabilized according to Foxp3 staining buffer kit instructions and then stained with direct flourochrome conjugated antibodies against ROR-γ, T-bet and GATA-3 in various combinations.

### Imaging flow cytometry

T helper cells were reactivated with anti-CD3 (1ug/ml plate bound) in presence or absence of MAPK inhibitors for 10 hours under standard cell culture condition. Intra/extra cellular proteins, including FasL, LAMP-1 were analyzed by Imaging flow cytometry on an Amnis I stream ISX machine with appropriate controls.

### MAPKs over expression and inhibitors studies on T helper cell death

Wild type MAPK plasmids such as Erk1, Erk2, JNK1a1 and p38α were transiently transfected with enhanced GFP as co-transfection marker as previously described [49] in Th17 like EL4 cells. PcDNA3.1 vector served as transfection control. After confirmation of transfection by flow cytometry, the transfected cells were activated with plate bound anti-CD3e for 16 hours and stained for Annexin-v APC. Propidium Iodide negative and eGFP positive cells were analyzed for Annexin-v binding.

MAPKs specific inhibitors such as p38 specific inhibitor SB203580, a MEK inhibitor PD98059 and a JNK inhibitor SP600125 were used to study MAPKs involvement in T helper cell AICD. A1.1 or Th1 and Th17 cells were pre incubated with MAPK inhibitors for 1 hour and reactivated with plate bound antiCD3e for 16 hrs. Optimal non-toxic concentration(s) of the inhibitors were determined and used in our studies.

### Immunostaining for activated MAPKs

T helper cells were reactivated in suspension with antiCD3e and cross-linked with secondary anti-Hamster IgG along with appropriate controls. Cell samples were collected at different time interval such as 0, 15, 30, 45, 60, 120, 180 and 240 minutes of post treatment. Further they were fixed with 4% paraformaldehyde for 20-30 minutes and washed in ice-cold 80% methanol for MAPK immuno staining as described [49]. Phospho p38, Erk1/2, JNK specific antibodies staining were performed and analyzed by flow cytometry. Isotype control served as background control at all time points.

### Detection of MAPKs and apoptotic proteins by western blotting

Secondary cross-linked T cells as mentioned above were used for western blot analysis. Briefly, cells were washed with ice-cold PBS, then re suspended in phosphosafe extraction buffer (Novagen, Merck Millipore, France) or a lysis buffer (20 mM Tris pH7.5, 150 mM NaCl, 1 mM EDTA, 1mM EGTA, 1% Triton X-100, 2.5 mM sodium pyrophosphate, 1mM b-glycerophosphate, 1mM Na_3_VO_4,_ 1mg/ml Leupeptin). The cell pellets were then sonicated 4 times for 5 seconds on ice and micro centrifuged for 10 minutes at 4°C. Proteins were quantified by 2D quant kit or Bradford reagent and separated by SDS-PAGE and probed with specific antibodies according to the manufacturer's instructions.

### Statistical analysis

Statistical significance for the presented data was assessed by Two-way ANOVA with Bonferroni post hoc test or Kruskal-Wallis test with Dunn's multiple comparison tests by using GraphPad Prism 5. Regression analysis was performed by using Sigma Plot 13.

## SUPPLEMENTARY MATERIALS



## Abbreviations

TCR-AICD: T Cell Receptor-Activation Induced Cell Death
MAPK's: Mitogen Activated Protein Kinases
TFs: Transcription Factors
TGF-β: Transforming Growth Factor beta
TNF-a: Tumor Necrosis Factor
IFN-g: Interferon-gamma
IL: Interleukin
Th0: unbiased T helper
Th1: T helper 1
Th17: T helper 17
