# Supplementary material for: Inherent low Erk and p38 activity reduce Fas Ligand expression and degranulation in T helper 17 cells leading to activation induced cell death resistance
Source: Oncotarget. 2016 Jul 28;7(34):54339–59. doi: 10.18632/oncotarget.10913 (PMC5342346; doi:10.18632/oncotarget.10913)
Supplement: Supplementary file 1 [file oncotarget-07-54339-s001.pdf]

Inherent low Erk and p38 activity reduce Fas Ligand expression and degranulation in T helper 17 cells leading to activation induced cell death resistance

Supplementary Material

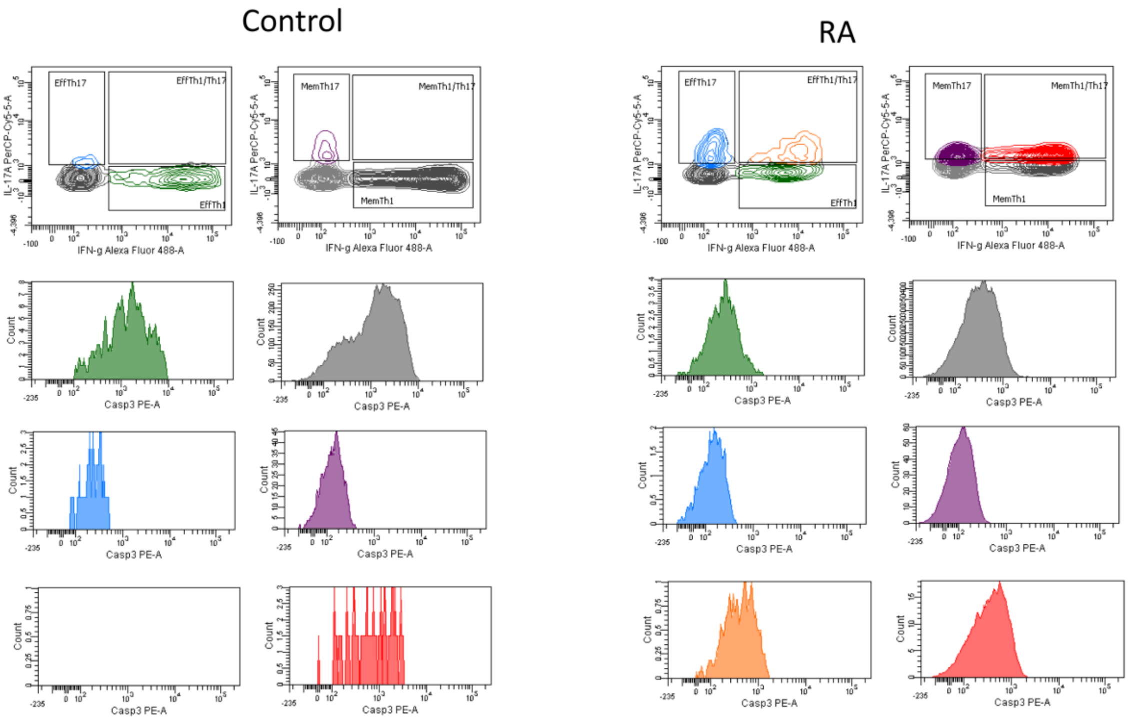

Supplement Figure.1.Characterization of RA and control T helper cells AICD:

Representative figure for detailed analysis of active caspase3 of Th1, Th17 and Th1/Th17 cells derived from effector (left) and memory (right) compartments in control (n=6) and RA (n=9).

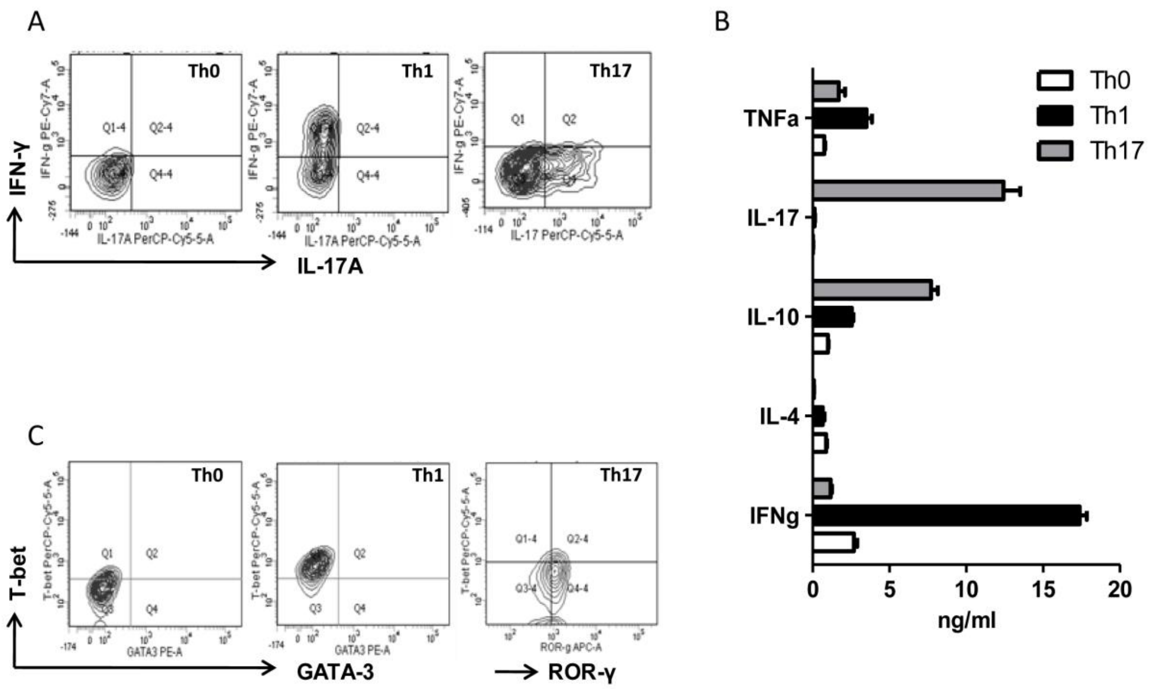

Supplement Figure.2.Characterization of in-vitro generated T helper cells: Representative figure for (A) intracellular cytokines and (C) transcription factors analysis of *in-vitro* generated mouse Th0, Th1 and Th17 cells. (B) Multiplex cytokine quantification from anti-CD3e stimulated mouse Th0, Th1 and Th17 culture supernatant.

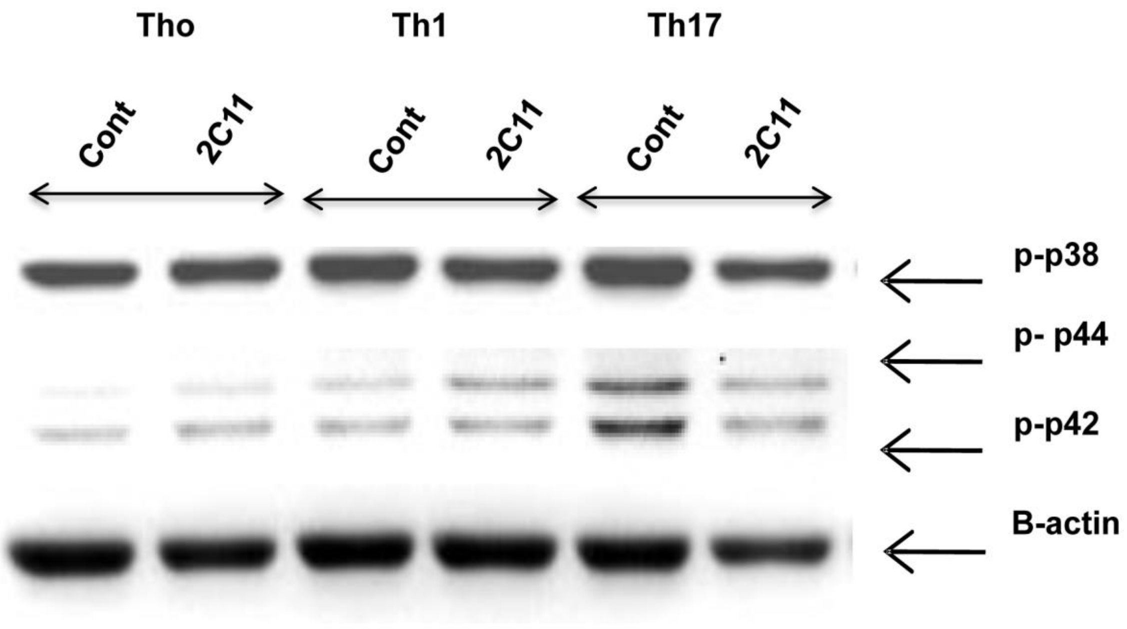

Supplement Figure.3.Western blot analysis of MAPK activity in T helper cells:

Representative Western Blot of phospho-p38, phospho-p44 and phospho-p42 with β- actin as loading control in secondary cross-linked mouse Th0, Th1 and Th17cells.

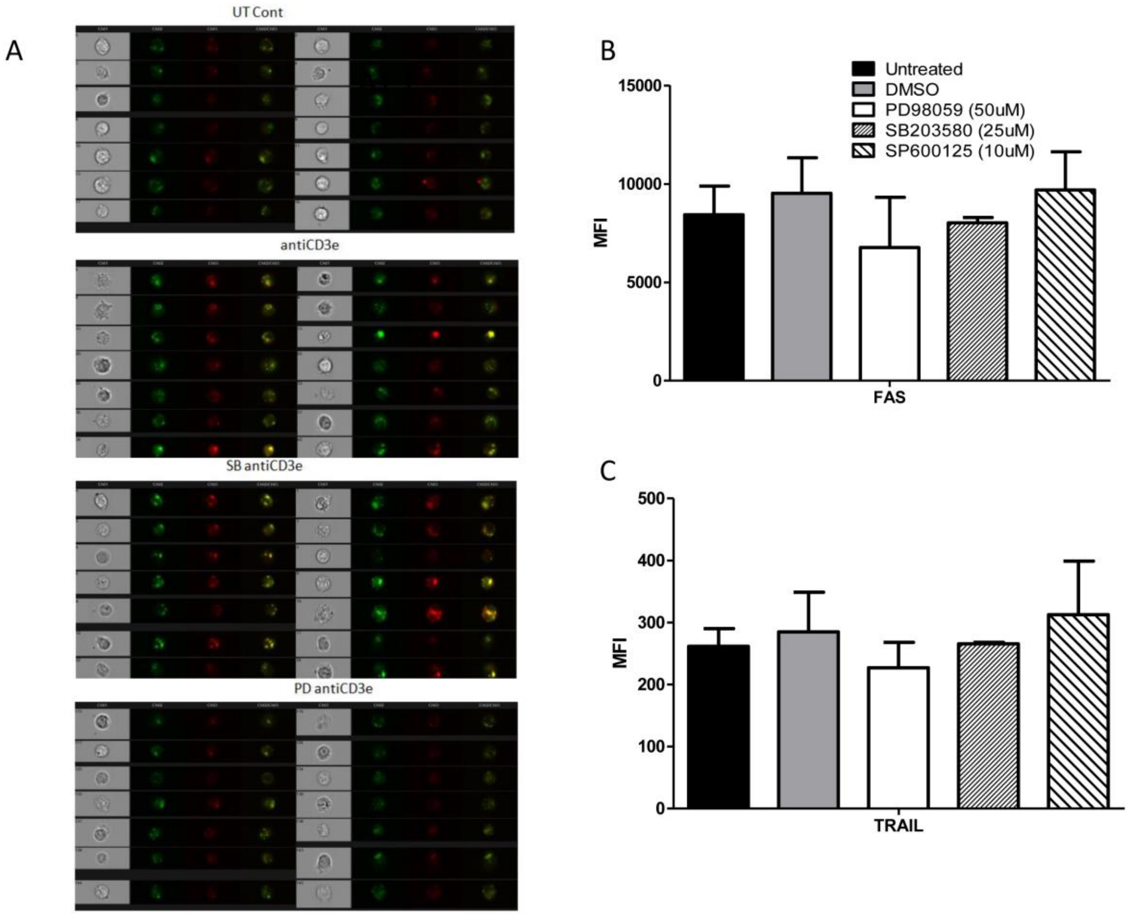

Supplement Figure.4. MAPK on FasL, Fas and Trail expression in T helper cells: A1.1 cells stimulated with anti-CD3e in the presence or absence of MAPK inhibitors were analyzed for (A) FasL (a representative imaging flow data), (B) Fas (Bar graph of Mean ± SEM) and (C) TRAIL (Bar graph of Mean ± SEM) expressions.

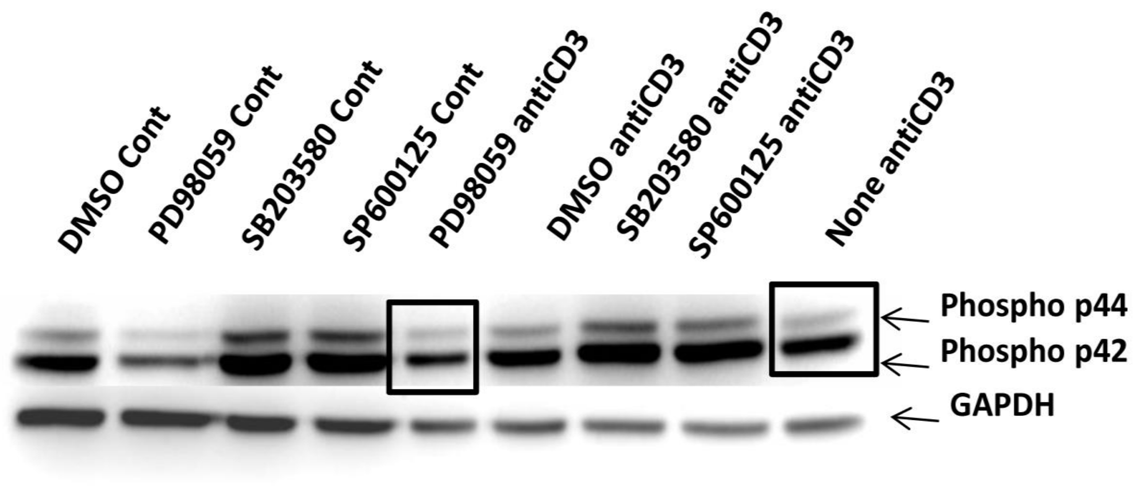

Supplement Figure.5. Specificity of MAPK inhibitors on Erk1/2 activity in T helper cells: **W**B analysis of phospho-p44 and phospho-p42 with GAPDH as loading control in secondary cross-linked A1.1 cells.

Table 1: Demographic data of Human RA and control subjects

	RA (n=27)	HC (n=15)
Age (yrs)	39.3 ± 8.3	30.5 ± 3.6
sex	M:4/F:23	M:5/F:10
ESR DAS score	5.89 ± 1.6	NA
Disease duration (month)	31.7 ± 32	NA
Drugs	DMARD	No drugs
